# Laboratory prediction of primary postpartum haemorrhage: a comparative cohort study

**DOI:** 10.1186/s12884-016-0805-3

**Published:** 2016-01-25

**Authors:** William K. B. A. Owiredu, Derick N. M. Osakunor, Cornelius A. Turpin, Osei Owusu-Afriyie

**Affiliations:** Department of Molecular Medicine, School of Medical Sciences (SMS), Kwame Nkrumah University of Science and Technology (KNUST), Kumasi, Ghana; Department of Obstetrics and Gynaecology, Komfo Anokye Teaching Hospital (KATH)/SMS, KNUST, Kumasi, Ghana

**Keywords:** Haemoglobin (anaemia), Laboratory, Postpartum haemorrhage, Prediction, Risk factors, Urea

## Abstract

**Background:**

Postpartum haemorrhage (PPH) is the leading cause of maternal deaths, the world over. The aim of this study was to determine laboratory parameters that could serve as risk factors for primary PPH.

**Methods:**

This comparative cohort study involved 350 pregnant women at term who were recruited consecutively from the Komfo Anokye Teaching Hospital, Kumasi, Ghana. PPH was defined as a measured blood loss ≥ 500 ml or enough to cause haemodynamic shock. Basic demographic data was gathered and blood was collected for laboratory assays before delivery. Univariate and multivariate logistic regression models were used to identify variables that were significantly associated with primary PPH.

**Results:**

Of the total recruited study participants (350), five declined to participate and 74 went through caesarean section, episiotomy or instrumental deliveries and were excluded. Of the remaining (271) study participants who went through spontaneous vaginal delivery, fifty five (55) were diagnosed with primary PPH (Group 1) and the remaining 216 were those who did not have PPH (Group 2). Demographic characteristics did not differ between the two groups (*P* > 0.05). Univariate analysis showed that AST (*P* = 0.043), urea (*P* < 0.001), creatinine (*P* = 0.002), urea-to-creatinine ratio (*P* = 0.014) and the proportion of abnormal peripheral blood smear (*P* < 0.001) was higher among women in Group 1 compared to those in Group 2. Women in Group 1 had a significantly lower haemoglobin concentration (10.7 g/dL) compared to those in Group 2 (12.1g/dL). Upon multivariate analysis, an abnormal peripheral blood smear (AOR = 2.9672), Hb, (AOR = 0.5791), moderate to severe anaemia (Hb <10 g/dL) (AOR = 3.1385), Urea (AOR = 3.6435) and intra-renal azotaemia (AOR = 0.1893) remained significant.

**Conclusion:**

Many laboratory parameters are associated with primary PPH but only a few are independent risk factors. A total clinical work-up including laboratory evaluation of the independent blood variables identified in this study will help a great deal to identify individuals at high risk for PPH.

## Background

Each year, about 289,000 women worldwide, die from various complications of pregnancy and childbirth and developing countries account for 99 % (286,000) of this rate [1]. In 2013, Ghana recorded a maternal mortality rate of 380 per 100,000 live births [2]. Among the various causes of maternal mortality, postpartum haemorrhage (PPH) has historically been the single most important [3].

Primary PPH is defined as a loss of 500 ml or more of blood (or any amount that causes haemodynamic compromise) during and after childbirth, within the first twenty-four hours [4]. The condition presents with several consequences, which could transform a healthy pregnant woman in labour to a critically ill patient [5], and efforts to save both mother and baby may pose serious challenges to health workers [6].

Although the burden of primary PPH is severe, only few studies in the developing world, have examined laboratory risk factors associated with primary PPH. Risk factors associated with primary PPH, as reported by other studies include non-white ethnicity [7], older age [8], history of PPH [9], history of blood disorders [7], nulliparity [10], low parity [11], grand multiparity [12], high blood pressure [13], antepartum haemorrhage [14] and multiple pregnancies [8].

However, many of these factors have a low predictive value [13]. Reports show that prediction of PPH with the current antenatal screenings is poor and about 60 % of cases have no identifiable risk [15, 16]. Other studies have shown that in women with three or more risk factors, predictive ability is as low as 10 % [17], with Tsu’s long-standing 1993 study reporting a prediction rate of below 7 % [18]. Furthermore, most reports on risk factors for PPH were conducted in the developed world, where conditions, facilities, expertise and even the experience of patients may vary from that in the developing world. This is supported by reports on the role of intrapartum factors - of which the above factors play a vital role, in the development of primary PPH [7, 9, 12, 13, 19, 20]. With the huge burden associated with primary PPH there is a need for continued examination of risk factors to provide better opportunities for prevention, intervention or active management in the early stages.

The main objective of this study was to determine laboratory parameters that could serve as risk factors for primary PPH. We hypothesized that various antepartum laboratory parameters will be associated with primary PPH and may be of value in the clinical management of pregnant women during labour.

## Methods

### Study design/Site

This was a comparative cohort study with a consecutive sampling technique, carried out from April to October 2012. Three hundred and fifty (350) women who had been admitted to the labour ward of the Department of Obstetrics and Gynaecology, Komfo Anokye Teaching Hospital (KATH), Kumasi, Ghana, were recruited for the study. This was done under the supervision of qualified midwives and Obstetrician/Gynaecologists.

### Eligibility

To qualify for recruitment, the pregnant women had to be on admission at the labour ward, with singleton pregnancy and should have consented to participate in the study. Women of all parity were included.

Women who underwent planned or emergency caesarean section, episiotomy and instrumental deliveries were excluded. Patients who bled after initial blood volumes had been estimated were also excluded. Women with pregnancy induced hypertension or pre-eclampsia, chronic metabolic diseases of which they were being treated or managed (e.g. diabetes mellitus, chronic kidney disease), hepatitis, HIV and tuberculosis were excluded. Inclusion was ascertained through review of patient folders in consultation with attending physicians.

### Participants

Three hundred and fifty (350) pregnant women who had been admitted to the labour ward of the Department of Obstetrics and Gynaecology for delivery were recruited as a start cohort. All recruited participants were followed up until delivery and an attending Obstetrician/Gynaecologist did an appropriate assessment of clinical status/diagnosis.

Five of the eligible recruits declined to participate in the study whereas 74 other patients underwent caesarean section, episiotomy or instrumental delivery or were lost to follow up. Of the 271 women who had normal vaginal delivery, 55 developed primary PPH (Group 1) and 216 did not develop primary PPH – normal vaginal delivery (Group 2).

### Sample and data collection

Prior to delivery, about 9 ml venous blood sample was collected from each participant into sodium citrate tubes for coagulation studies (Prothrombin Time - PT and activated Partial Thromboplastin Time - aPTT), serum separator tubes for biochemical assays (Total protein- TP, Albumin - ALB, Globulins-GLOB, Alanine Aminotransferase - ALT, Aspartate Aminotransferase - AST, urea -URE and creatinine - CRE) and ethylenediaminetetraacetic acid tubes (EDTA) tubes for haematological assays (Full Blood Count - FBC, Erythrocyte Sedimentation Rate - ESR, Glucose-6-phosphate Dehydrogenase - G6PD, sickling, blood group and peripheral blood smear).

Haematological assays were performed on fresh anticoagulated blood using an automated analyser (Mindray BC 3000 Plus, Shenzhen, China). Serum separator and sodium citrated samples were centrifuged and aliquots of plasma or serum were stored at −80°C until assayed. Biochemical assays were performed using an automated analyser (Selectra Pro S, Vital Scientific NV, Netherlands). Coagulation parameters were assayed using a semi-automated analyser (Coatron M4 TECO Medical Instruments, Germany). All other parameters were gathered from the patient records.

### Measurement of blood loss

Deliveries were done on a dry disposable “linen saver” as suggested in a similar study [21]. The amniotic fluid was allowed to drain away and blood was allowed to collect into a graduated receptacle placed below the delivery bed, after which a qualified Midwife measured the amount of blood loss. Diagnosis of primary PPH (by an attending physician-Obstetrician/Gynaecologist) was based on vaginal delivery with a measured blood loss of 500 ml or more, up to one hour post-delivery. Vaginal delivery with a measured blood loss of less than 500 ml but enough to cause signs of haemodynamic compromise (e.g. weakness, syncope, decreased level of consciousness, dizziness, chest discomfort, dyspnoea, hypotension diaphoresis and pulmonary congestion) also constituted primary PPH [4, 5, 8, 22].

### Outcome criteria

All laboratory results were classified into various categories based on reference ranges with respect to pregnancy to accommodate the physiological changes [23].

In the peripheral blood smear, any feature consistent with the presence of abnormal number, shape, size and age of cells constituted an abnormal peripheral blood smear.

Classification of anaemia was as per the recommendations of the World Health Organization [24]. Azotaemia was classified as pre-renal when urea: creatinine ratio was > 100, post-renal when ratio was from 40–100 and intra-renal when ratio was < 40.

### Statistics

Data was entered into a Microsoft Excel spreadsheet and statistical analysis was done using the R-console (The R foundation for statistical computing, http://www.R-project.org). We reported categorical variables as frequencies and proportions and compared them with Chi-square and Fisher’s exact test as appropriate. We reported continuous variables as mean (SD) or median (IQR) based on data distribution (normal or skewed respectively) and compared them using Wilcoxon-Mann–Whitney (Rank sum) test and Independent samples *t*-test for skewed and normally distributed data respectively. Multivariate logistic regression analysis was done to identify independent risk factors associated with primary PPH. Independent risk factors were identified using an automatic selection process (MODELSTEP), which removes non-significant variables and confounders in a stepwise backward selection process, based on a likelihood ratio static. An Akaike’s Information Criterion (AIC) is constantly generated and the last MODELSTEP with the smallest AIC was selected as the best-fit model. Due to the low numbers of some blood types observed, this variable was categorised into “Blood type O” and “non-O blood types”.

### Ethical Consent

The study was approved by the Committee on Human Research Publication and Ethics (CHRPE) of the Kwame Nkrumah University of Science and Technology (KNUST)/School of Medical Sciences (SMS) as well as the Research and Development unit of the KATH. The objective of the study was explained to all participants and written informed consent was obtained before recruitment.

## Results

Of the total number of participants approached (350), five declined to participate and a further 74 were excluded from data analysis and presentation due to selection criteria used in the present study. Of the remaining 271 study participants who went through spontaneous vaginal delivery, fifty five (55) were diagnosed with primary PPH (Group 1) where as two hundred and sixteen (216) did not present with any such complication (Group 2).

The mean age of participants was 27.7 ± 5.7 years and Groups 1 and 2 did not differ based on age, BMI, parity, gravidity, history of antepartum bleeding, induced and spontaneous abortions, transfusions, contraception, fibroids and surgery (*P* > 0.05).

Table 1 shows a list of biochemical and coagulation parameters of the study population. AST (*P* = 0.043), urea (*P* < 0.001), creatinine (*P* = 0.002) and Urea-to-creatinine ratio (*P* = 0.014) levels were higher amongst women in Group 1 than in Group 2.

**Table 1:** Biochemical and coagulation parameters of study participants

Parameters	All women (*n* = 271)	Group 1 (*n* = 55)	Group 2 (*n* = 216)	*P* value
AST *median (IQR)*	17 (13.5,21)	18 (15,22.5)	16 (13,20)	0.043
ALT *median (IQR)*	9 (6,11)	8 (6.5,11)	9 (6,11)	0.48
TP *median (IQR)*	68 (63,73)	66 (59.5,74)	68 (63,73)	0.315
ALB *median (IQR)*	36 (34,38)	35 (31.5,38)	37 (35,39)	0.001
GLOB *median (IQR)*	30 (28,34)	31 (27.5,36)	30 (28,34)	0.889
URE *median (IQR)*	1.8 (1.5,2.4)	2.5 (1.8,3.2)	1.8 (1.5,2.3)	<0.001
CRE *median (IQR)*	66 (52.5,78.5)	72 (61,90.5)	61.5 (51,75.2)	0.002
URE: CRE				
Median *(IQR)*	28 (22,38)	31 (24,45)	27 (21,36.8)	0.014
Intra-renal azotaemia *n (%)*	217 (80.7)	35 (63.6)	182 (85)	<0.001
Normal *n (%)*	50 (18.6)	18 (32.7)	32 (15)	
Pre-renal azotaemia *n (%)*	2 (0.7)	2 (3.6)	0 (0)	
PT *median (IQR)*	13 (12,15)	14 (13,16)	13 (12,15)	0.096
INR *median (IQR)*	1.1 (1,1.3)	1.2 (1.1,1.4)	1.1 (1,1.3)	0.096
aPTT *median (IQR)*	28 (24,36)	31 (24,38)	28 (24.8,33.5)	0.576

Data are presented as *n* (%) or median (IQR). All P values less than 0.05 were considered significant. Group 1: Women with primary PPH. Group 2: Women without primary PPH. IQR: Inter-quartile range. n: Number. AST: Aspartate Transaminase. ALT: Alanine Transaminases. TP: Total Protein. ALB: Albumin. GLOB: Globulins. URE: Urea. CRE: Creatinine. PT: Prothrombin Time. aPTT: Activated Partial Thromboplastin Time. INR: International Normalised Ratio

Women in Group 1 had a significantly lower Hb (10.7 g/dL) compared to 12.1 g/dL in Group 2 (*P* < 0.001). A larger proportion of Group 1 participants had moderate/severe anaemia in addition to an abnormal peripheral blood smear, than in Group 2 (*P* < 0.001 each). Total WBC, NEUT, MONO and ESR levels were higher amongst women in Group 1 compared to Group 2 (*P* < 0.05). Laboratory-confirmed presence of malaria parasitaemia was low (6.3 %) in the total population. [Tables 2 and 3]

**Table 2:** Haematological parameters of study participants

Parameters	All women (*n* = 271)	Group 1 (*n* = 55)	Group 2 (*n* = 216)	*P* value
Hb (g/dL) *median (IQR)*	11.9 (10.9,12.8)	10.7 (8.5,12.3)	12.1 (11.3,13)	<0.001
ANAEMIA *n (%)*				
Mod-Sev	36 (13.3)	22 (40)	14 (6.5)	<0.001
Mild	37 (13.7)	9 (16.4)	28 (13)	
Normal	198 (73.1)	24 (43.6)	174 (80.6)	
G6PD *n (%)*				
Full Defect	12 (4.4)	0 (0)	12 (5.6)	0.169
No Defect	257 (94.8)	55 (100)	202 (93.5)	
Partial Defect	2 (0.7)	0 (0)	2 (0.9)	
SICKLING *n (%)*				
Negative	235 (86.7)	47 (85.5)	188 (87)	0.931
Positive	36 (13.3)	8 (14.5)	28 (13)	
BLOOD GROUP *n (%)*				
Non-O groups	130 (47.9)	22 (40)	108 (50)	0.227
Group O	141 (52.1)	33 (60)	108 (50)	
HCT *median (IQR)*	36.1 (32.9,38.5)	32.1 (25.2,36.8)	36.5 (33.6,38.5)	<0.001
MCV *median (IQR)*	84.8 (80.5,88.6)	85.9 (81.6,88.2)	84 (80.1,88.7)	0.426
MCH *median (IQR)*	28.1 (26.9,29.7)	29 (27.4,30.1)	28.1 (26.8,29.6)	0.151
MCHC *median (IQR)*	33.2 (32.6,34.3)	33.4 (32.8,34.3)	33.2 (32.6,34.3)	0.248
PLT *mean (SD)*	205.8 (66.8)	210 (69.3)	204.7 (66.2)	0.597

Data are presented as n (%) or median (IQR) or mean (SD). All *P* values less than 0.05 were considered significant. Group 1: Women with primary PPH. Group 2: Women without primary PPH. IQR: Inter-quartile range. n: Number. Mod: Moderate. Sev: Severe. Hb: Haemoglobin. G6PD: Glucose-6-Phosphate Dehydrogenase. HCT: Haematocrit. MCV: Mean Cell Volume. MCH: Mean cell haemoglobin. MCHC: Mean Corpuscular Haemoglobin Concentration. PLT: Platelets

**Table 3:** Haematological parameters of study participants

Parameters	All women (*n* = 271)	Group 1 (*n* = 55)	Group 2 (*n* = 216)	*P* value
WBC *median (IQR)*				
Total	9.4 (7.4,12.9)	10.2 (8.8,15)	9.4 (7.1,12.5)	0.023
NEUT	7.3 (5.1,11)	8.6 (6,12.9)	7 (4.8,9.8)	0.004
LYMPH	1.5 (1.2,2)	1.6 (1.2,1.9)	1.5 (1.2,2)	0.831
MONO	0.7 (0.6,0.9)	0.8 (0.5,1.2)	0.7 (0.6,0.9)	0.017
EO	0 (0,0.1)	0 (0,0.1)	0 (0,0.1)	0.116
BASO	0 (0,0)	0 (0,0)	0 (0,0)	0.119
ESR (mm/hr) *median (IQR)*	56 (39.5,76)	70 (48,96)	55 (37,75)	0.018
MALARIA PARASITAEMIA *n (%)*				
Negative	254 (93.7)	51 (92.7)	203 (94)	0.756
Positive	17 (6.3)	4 (7.3)	13 (6)	
PERIPHERAL BLOOD SMEAR *n (%)*				
Abnormal	99 (36.5)	39 (70.9)	60 (27.8)	<0.001
Normal	172 (63.5)	16 (29.1)	156 (72.2)	

Data are presented as n (%) or median (IQR) or mean (SD). All P values less than 0.05 were considered significant. Group 1: Women with primary PPH. Group 2: Women without primary PPH. IQR: Inter-quartile range. N: Number. WBC; White Blood Cell. NEUT: neutrophils. LYMPH: Lymphocyte. MONO: Monocytes. EO: Eosinophil. BASO: Basophil. ESR: Erythrocyte Sedimentation Rate

Upon multivariate analysis, five variables remained significant. An abnormal peripheral blood smear was associated with the development of primary PPH (AOR = 2.9672). For every unit increase in Hb, the odds of developing primary PPH decreased 0.5791 times (95 % CI = 0.4585 to 0.7314) and women with moderate to severe anaemia (Hb <10 g/dL) were three times more likely to develop primary PPH than women with no or mild anaemia. For every unit increase in serum urea, the odds of primary PPH increased 3.6435 times. Intra-renal azotaemia was associated with a less likelihood of developing primary PPH (AOR = 0.1893, 95 % CI = 0.0602 to 0.5955) compared to pre-renal or post-renal azotaemia. [Table 4]

**Table 4:** Multivariate analysis for independent factors associated with primary PPH

Parameter	Adj OR	95 % CI	*P* value
Peripheral blood smear			
*Normal			
Abnormal	2.9672	1.3323 to 6.6083	0.0078
Hb	0.5791	0.4585 to 0.7314	<0.0001
Anaemia			
*Normal			
Mod-severe	3.1385	1.2099 to 8.1412	0.0187
URE: CRE			
* Normal			
Intrarenal azotaemia	0.1893	0.0602 to 0.5955	0.0044
UREA	3.6435	2.0968 to 6.3311	<0.0001

CRE: Creatinine. URE: Urea. OR: Odds ratio. Adj: Adjusted. CI: Confidence Interval. P values less <0.05 were considered significant. * Reference variable

## Discussion

We hypothesized that various antepartum laboratory parameters will be associated with primary PPH and will have the ability for identification of high-risk groups. The results of our study confirm this hypothesis but only few of the parameters included in the present study showed independent association. A number of factors showed an association with primary PPH; AST, urea, creatinine, urea-to-creatinine ratio, haemoglobin concentration, WBC, ESR and the state of a peripheral blood smear (normal or abnormal). Multivariate analysis showed that increasing urea and urea-to-creatinine ratio, decreasing haemoglobin concentration (<10 g/dL) and an abnormal peripheral blood smear were independently associated with a higher likelihood of developing primary PPH.

### Biochemical parameters and primary PPH

AST and ALB levels, although differed significantly between the two groups, were within normal limits of pregnancy [23]. This is similar to a study where there was significant changes in ALT but not in other liver enzymes [25]. Higher levels of AST than ALT could be accounted for from other sources of the enzyme, the heart, skeletal muscles, kidney, brain and red cells [26]. The exclusion of participants with liver infection and other chronic liver diseases could be a contributing factor for the observation of normal liver function markers amongst the study participants.

Urea and creatinine levels were higher amongst Group 1 (women with primary PPH) than in Group 2. Creatinine is expected to decline in pregnancy [23, 27], hence mild elevations during pregnancy may signify mild renal insufficiency, as opposed to that in non-pregnant women. We however did not determine the estimated glomerular filtration rate (eGFR) of participants, therefore this suggestion is not conclusive. Upon multivariate analysis, Urea and urea-to-creatinine ratio remained significant associations of primary PPH. It has been suggested that excess urea can cause an upregulation of endothelial nitric oxide through shunting of L-arginine to form guanidinosuccinic acid [28]. Consequently, this causes reduced endothelial adhesion of platelets due to impaired Von-Willibrand Factor (VWF) binding to platelets, therefore higher bleeding tendencies [28].

### Coagulopathy and primary PPH

Measures of coagulation, PT and aPTT did not differ between the two groups and hence showed no significant association. It is expected that bleeding tendencies will correlate with abnormalities in coagulation markers. Although the current study could not demonstrate this the finding is still in line with findings that coagulation abnormalities contribute only about 1 % of the causes of PPH [29], hence our observation. It is important however, to note that coagulation abnormalities cannot be ruled out. Suggestions are that a hypercoagulable state results in the second and third trimesters due to an increased synthesis and activity of coagulation factors − fibrinogen, coagulation factors (F) VII, VIII, IX, X, XII, and VWF − and results in a shortened PT and aPTT [30]. This could be a contributing factor to our observation of a pseudo-normal PT and aPTT. Hence coagulation abnormalities may have been present but masked by physiological changes, and may be apparent only after pregnancy.

### Haematological parameters and primary PPH

We found that increasing haemoglobin concentrations were associated with a less likelihood of primary PPH and that women with [Hb] < 10 (moderate-severe anaemia) were more likely to develop primary PPH. Anaemia is responsible for 17–46 % of cases of maternal deaths when combined with obstetric haemorrhage [22]. In addition, complications of PPH, pre-term delivery and foetal growth restriction are more frequent in anaemic women [31]. In a similar study, women in Zimbabwe with an [Hb] of less than 12 g/dL were 2.2 times more likely to develop PPH than non-PPH controls [18]. Researchers in Tanzania reported that [Hb] of < 9 g/dL was strongly associated with bleeding in the immediate postpartum period [12]. In contrast to our findings, two previous studies reported no association between anaemia and PPH in Nigeria [32] and no difference in risk of postpartum haemorrhage between severe and moderate anaemia in Ghana [33]. These studies however relied on visual estimation, a technique known to underestimate blood loss [5, 34].

It has been hypothesized that severe anaemia may weaken uterine muscles or lower resistance to infectious diseases thus leading to PPH [35, 36]. Furthermore, decreased uterine blood flow or low uterine muscle strength may contribute to inefficient uterine contractions and contribute to blood loss, potentially mediated by iron-deficiency anaemia [37, 38]. The findings of this current study have demonstrated that the severity of anaemia places a woman at a greater risk of experiencing PPH. It also confirms the hypothesis that severe anaemia may impair the tolerance of women to the effects of PPH due to their inability to endure excessive blood losses [39, 40].

The association of low haemoglobin concentrations and thus anaemia with primary PPH is consistent with our observation that an abnormal blood film was associated with a higher risk of primary PPH. It is noteworthy that the morphological appearance and number of red cells are largely affected by the haemoglobin concentration and anaemia. Furthermore, other conditions that may affect platelet function and numbers leading to an abnormal peripheral blood smear may stimulate bleeding thus leading to PPH.

### Recommendations and impact on health delivery system

Currently in Ghana, the routine antenatal laboratory tests and screening include some but not all the parameters and variables identified in the present study. While we have made progress, the current interventions are inadequate. The practicality of carrying out the extra tests proposed in the present study, including cost, capacity of the facility and the laboratory, as well as cost implications to the patient and the facility should be evaluated appropriately by the relevant authority. Accordingly, a review of antenatal policies to cater for such additional proposed tests may be warranted.

A total clinical work-up including laboratory evaluation of the independent blood variables identified in this study could help in identifying individuals at high risk or otherwise for PPH. The will contribute to prevention, intervention or ensuring preparations for active management in the early stages.

In settings where there is lack of skilled personnel, a more preventive approach to primary PPH is recommended; indicators identified in the present study could be used to identify and refer high-risk individuals to better-resourced facilities to prevent maternal and neonatal death in the event of the occurrence of primary PPH.

## Conclusions

Many laboratory parameters are associated with primary PPH, However, only a few serve as independent risk factors; increasing urea and urea-to-creatinine ratio, decreasing haemoglobin concentration (<10 g/dL) and an abnormal peripheral blood smear were independently associated with a higher likelihood of developing primary PPH.

### Limitations

There is a limitation on the generalization of the study findings due to the consecutive sampling technique and the relatively smaller sample size used. Further studies using a larger sample pool in matched case–control studies are warranted. Furthermore, a representation of haemoglobin levels and the extent of blood loss in each group would better buttress the relationship between Hb and the extent of blood loss required for primary PPH development. However given the lack of availability of such data in Ghana, findings from this study however remain relevant and add to evidence on the subject matter.

## Abbreviations

ALB: Albumin
ALT: Alanine Aminotransferase
AMSTL: Active Management of the Third Stage of Labour
aPTT: Activated Partial Thromboplastin Time
AST: Aspartate Aminotransferase
CRE: Creatinine
EDTA: ethylene-diamine-tetra-acetic acid
ESR: Erythrocyte Sedimentation Rate
FBC: Full Blood Count
G6PD: Glucose-6-phosphate Dehydrogenase
GLOB: Globulins
KATH: Komfo Anokye Teaching Hospital
MDG: Millennium Development Goals
PPH: Postpartum haemorrhage
PT: Prothrombin time
TP: Total Protein
URE: urea
VWF: Von-Willibrand Factor
